# Population pharmacokinetic model development and its relationship with adverse events of oxcarbazepine in adult patients with epilepsy

**DOI:** 10.1038/s41598-021-85920-0

**Published:** 2021-03-18

**Authors:** Yoonhyuk Jang, Seonghae Yoon, Tae-Joon Kim, SeungHwan Lee, Kyung-Sang Yu, In-Jin Jang, Kon Chu, Sang Kun Lee

**Affiliations:** 1 Department of Neurology, Laboratory for Neurotherapeutics, Comprehensive Epilepsy Center, Biomedical Research Institute, Seoul National University Hospital, 101 Daehak-ro, Jongno-gu, Seoul, 110-744 South Korea; 2 Department of Clinical Pharmacology and Therapeutics, Seoul National University Bundang Hospital, Seongnam, Korea; 3 Department of Clinical Pharmacology and Therapeutics, Seoul National University College of Medicine and Hospital, Seoul, Korea; 4 Department of Neurology, Ajou University School of Medicine, Suwon, South Korea

**Keywords:** Epilepsy, Molecular medicine

## Abstract

This study aimed to develop a pharmacokinetic (PK) model of oxcarbazepine (OXC) and analyse the relationship between monohydroxylated derivative (MHD), an active metabolite of OXC, and the adverse events of OXC. We obtained 711 OXC samples from 618 patients with epilepsy who were enrolled in the Epilepsy Registry Cohort of Seoul National University Hospital from February 2011 to January 2014. The plasma PK model was developed using a nonlinear mixed-effect modelling method with NONMEM (ver 7.3). A one-compartment model with a first-order absorption model and proportional residual error adequately described the MHD concentration–time profiles. The only covariate incorporated for CL/F and V/F was body weight. Of the 447 patients analysed, 28 (6.26%) had dose-related adverse events (DRAEs), which were dizziness, somnolence, headache, and diplopia. For DRAE occurrence, the cut-off values of the MHD trough and AUC were 12.27 mg/L (specificity 0.570, sensitivity 0.643) and 698.5 mg h/L (specificity, sensitivity 0.571), respectively. Multivariate analysis showed the sole dizziness symptom was significantly associated with both the MHD trough and the AUC (*p* = 0.013, *p* = 0.038, respectively). We newly developed a population PK model using sparse sampling data from patients with epilepsy, and the model better reflects the actual clinical situation.

## Introduction

Oxcarbazepine (OXC) is an antiseizure medication (ASM) that is prevalently prescribed for patients with epilepsy. OXC, as a structural variation form of carbamazepine (CBZ), is an effective option for focal seizures as a monotherapy or adjuvant therapy and has fewer adverse events than CBZ^1–3^.

OXC is rapidly absorbed and almost completely converted to its active metabolite 10,11-dihydro-10-hydroxy-carbamazepine (monohydroxy derivative, MHD), which has a major role in the antiseizure effect in humans. Whereas only 2% of total radioactivity in plasma is due to unchanged OXC, more than 70% is attributed to MHD metabolized by cytosolic aryl ketone reductase in the liver^4–6^. Thus, for patients who take OXC, exposure to MHD is approximately 15 times higher than exposure to OXC^7^. OXC and MHD exert their antiseizure activity by blocking voltage-sensitive sodium channels and stabilizing hyperexcited neuronal membranes^8^. OXC is cleared mostly in the form of metabolites, which are excreted by the kidneys. In addition, OXC is known to have drug-drug interactions, acting as a perpetrator and substrate at the same time. Several studies suggest that cytochrome P450 (CYP) isoenzyme-inducing drugs (e.g., phenytoin, phenobarbital, carbamazepine) decrease the concentration of MHD, which may require an increase in the OXC dose to maintain drug effects^6,9,10^.

Serum MHD levels also play a critical role in the occurrence of adverse events induced by OXC. The adverse events of OXC include dizziness, diplopia, ataxia, skin rash, and hyponatremia^3,11^, and the different pathomechanisms are known to be associated with adverse events. Maculopapular eruption, which is one of the most serious events of OXC, is associated with autoimmune reactions involving specific subtypes of human leukocyte antigen^12^. In contrast, dizziness, the most common adverse event of OXC, is dependent on MHD serum levels^13^, implying that pharmacokinetics (PK) of OXC may play an important role. Additionally, dose-related adverse events (DRAEs) are known to include dizziness, somnolence, headache, and diplopia^3^; however, the specific cut-off levels or PK variables of OXC and MHD that cause DRAEs have not been studied previously.

In this study, we developed a population PK model of MHD to explore various covariates, including comedications. We also investigated adverse events related to OXC and the relationship between PK and these adverse events.

## Methods

### Patient enrolment, samples and assays

We obtained 711 OXC samples from 618 patients with epilepsy who were enrolled in the Epilepsy Registry Cohort of Seoul National University Hospital from February 2011 to January 2014. All spot samples were collected from patients who were each taking the same dose of ASMs including oxcarbazepine for at least a month and the drug level was assumed as a steady-state. Serial blood samples were obtained from the single-dose study at 2, 4, 6, 8, 12, 14, 16, and 24 h post dose. The actual dosing and sampling time were used in the analysis. Plasma concentrations of MHD were determined using the validated liquid chromatography-tandem mass spectrometry method^7^.

Variables including body weight, final dosing time, and blood sampling time were obtained and available in 447 patients whose data were used for further analysis. Among the 447 patients, most of them (n = 438) received OXC twice a day, five received once, and four received three times a day. To develop a PK model, the data of the PK study involving 40 patients evaluating a 30 mg/kg single oral dose of OXC were combined^7^. The retrospective review was performed from each patient’s medical records, including OXC dosing histories, times of blood sampling, seizure frequency, reported adverse events, coadministered ASMs and their regimen, and routine laboratory results. Seizure-free was defined as having no epileptic seizure for at least three months before the blood sample was taken. The existence of dizziness, somnolence, diplopia, tremor, ataxia, dysarthria, encephalopathy, cerebral atrophy, psychiatric symptoms, fatigue, headache, visual field defect, epistaxis, skin rash, haematologic effects, liver function test abnormalities, hyponatremia, reproductive problems, cardiovascular complications, gastrointestinal problems, body weight changes, paresthesia, renal stone, osteoporosis, acne, hirsutism, hair loss, gingival hyperplasia, and limb oedema was checked as adverse events of OXC administration. The criteria for considering OXC-induced adverse events were as follows: Symptoms that complained very frequently after starting OXC or after increasing the dose, and symptoms rarely complained before. Two neurology specialists (Kon Chu and Sang Kun Lee) with long (> 20 years) clinical experience were in charge of all the patients in this study, and checked for side effects including dizziness at the epilepsy clinic.

Among them, dizziness, somnolence, headache, and diplopia were defined as DRAEs^3^. Hyponatremia, which was failed to verify the association with OXC dose in some references, was not included as DRAE^11,14^. Dizziness related to epilepsy itself was differentiated with OXC-induced dizziness according to the baseline frequency of the seizure and the habitual symptom.

This study was approved by the Institutional Review Board of Seoul National University (IRB No. 1010-042-335, 12/06/2010). Written informed consent was obtained from all patients. All methods were performed in accordance with the relevant guidelines and regulations. Informed consent was obtained for all the subjects who are under 18, from a parent and/or legal guardian.

### Population PK model development and model evaluation

We developed a population PK model of MHD using a nonlinear mixed-effects modelling method with NONMEM, version 7.3. A total of 748 data points of 487 patients were used in this analysis. We assumed that MHD followed a one-compartment model with first-order elimination, which was also adopted in other studies. The ADVAN2 subroutine and first-order conditional estimation with the interaction method were used. The absorption rate (ka), apparent clearance (CL/F) and apparent volume of distribution (V/F) were estimated. The interindividual variability (IIV) associated with PK parameters was modelled using an exponential model as follows:

$$\theta_{i} = \theta_{typical} \times e^{{\eta_{i} }}$$

where θ_i_ is the parameter for the ith subject, θ_typical_ is the typical value of the parameter, and η_i_ is a normally distributed random variable for the ith subject, with a mean of zero and variance ω^2^. Several error models, including additive, proportional, and combined error models, were tested, and a proportional error model was used in the final model. The NONMEM code for the final model can be found in Supplementary material.

The effects of covariates such as age, sex, and concomitant drugs were explored. Body weight was incorporated as a covariate for CL/F and Vd considering its relationship with PK parameters^15–17^. The effects of other ASMs (phenytoin, valproate, phenobarbital, lamotrigine, pregabalin, topiramate, zonisamide, levetiracetam, clobazan, vigabatrin, lorazepam) or comedication with enzyme-inducing ASMs (EIASMs; carbamazepine, phenytoin, or phenobarbital) were evaluated. Stepwise forward selection and a backward elimination method were used. The objective function value (OFV) decrease of 3.84 (χ^2^ distribution, *p* < 0.05) was considered significant in the forward selection process; the OFV increase of 6.63 (*p* < 0.01) was considered significant in the backward elimination process.

The adequacy of each model was evaluated based on not only OFV but also goodness-of-fit plots. Plots of observed versus predicted values of individual and population values were evaluated for randomness around the line of unity. Plots of conditional weighted residual (CWRES) versus population predicted values and time after dose were evaluated for randomness around the zero line. To evaluate the stability and robustness of the final PK model, a bootstrap resampling method was used. Resampling with replacement generated 1,000 bootstrap datasets, and the final population PK model was fitted to each of these datasets. The median and 95% confidence intervals of parameters obtained from this step were compared with the final parameter estimates. A visual prediction check was also performed using a graphical comparison of the simulated data overlaid with the observed data.

The apparent clearance (CL/F) of each individual was estimated from the final model and AUC was calculated using the following equation: AUC = Dose/(CL/F). The trough concentration of MHD was estimated using a simulation method. The dose used for the calculation was the dose actually taken by each patient.

### Statistics

Clinical data are presented as the mean ± standard deviation or number with percentage. For group comparisons, we utilized Mann–Whitney U tests or Fisher’s exact tests for continuous or categorical variables. The difference in PK variables between the groups with and without adverse events was analysed with the Wilcoxon matched pairs signed-rank test. The optimal cut-off value of the MHD levels for the prediction of adverse events was identified using the receiver operating characteristic (ROC) curve (R package pROC and a web-tool for ROC curve analysis). In the ROC curve, the cut-off value was calculated as the point where Youden's index (sensitivity + specificity − 1) became the maximum^18^. R version 4.0.2 (R Foundation for Statistical Computing, Vienna, Austria) was used for analysis, and a *p*-value < 0.05 was considered statistically significant.

### Ethical statement

We confirm that we have read the journal’s position on issues involved in ethical publication and affirm that this report is consistent with those guidelines.

## Results

### Patient characteristics

Of the 447 patients analysed, two hundred sixty-four (59.1%) were male (Table 1). The mean age was 39.2 years (range 16 to 80), and the average body weight was 65.8 kg (range 39 to 116). The number of patients who were administered the combination therapy was 292 (65.3%), and on average, they took two ASMs, including OXC (interquartile 1 to 3). The most common coadministered ASM was levetiracetam (162, 55.5%), followed by topiramate (96, 32.9%). Among the patients taking multiple ASMs, only 23 (5.1%) had EIASMs. In total, 63.8% (285) of the patients were seizure-free, and the daily OXC dose was 999 mg on average (range 150 to 2100).

**Table 1:** Characteristics of patients taking oxcarbazepine according to the occurrence of dose-related adverse events.

Parameter	Total (N = 447)	Dose-related adverse events			Only dizziness		
		AE+ (n = 28)	AE- (n = 419)	*P* value	Dz+ (n = 18)	Dz- (n = 429)	*P* value
**Demographic**							
Age (yrs)	39.2 ± 14.7	37.3 ± 14.8	39.3 ± 14.7	0.449	34.9 ± 13.1	39.4 ± 14.7	0.211
Sex, male	264 (59.1%)	14 (50.0%)	250 (59.7%)	0.327	10 (55.6%)	254 (59.2%)	0.809
Body weights (kg)	65.8 ± 12.5	63.7 ± 12.2	65.9 ± 12.5	0.489	68.2 ± 9.6	65.7 ± 12.6	0.235
**Clinical information**							
No. of ASMs^a^	2 [1–3]	2 [1–3]	2 [1–3]	0.375	2 [1–3]	2 [1–3]	0.950
Co-ASMs	292 (65.3%)	19 (67.9%)	273 (65.2%)	0.840	11 (61.1%)	281 (65.5%)	0.801
Co-EIASMs	23 (5.1%)	2 (7.1%)	21 (5.0%)	0.648	0	23 (5.4%)	0.614
Seizure-free	285 (63.8%)	13 (46.4%)	272 (64.9%)	0.066	8 (44.4%)	277 (64.6%)	0.130
**OXC PK**							
OXC dose (mg)	999 ± 338	1130 ± 512	990 ± 322	0.181	1216 ± 598	989 ± 321	0.053
OXC dose/WT (mg/kg)	15.4 ± 0.24	18.0 ± 1.37	15.3 ± 0.24	0.064	17.8 ± 1.84	15.3 ± 0.24	0.248
OXC level (mg/L)	721 ± 38.8	915 ± 150	709 ± 40.2	0.179	981 ± 208	711 ± 39.5	0.359
MHD level (mg/L)	15,756 ± 324	17,080 ± 1255	15,668 ± 336	0.175	16,449 ± 1863	15,728 ± 329	0.595
MHD trough level (mg/L)	12.75 ± 7.17	15.24 ± 8.26	12.58 ± 7.08	0.086	17.48 ± 8.89	12.55 ± 7.04	0.013*
MHD AUC (mg∙h/L)	653.8 ± 268.7	753.1 ± 285.3	647.2 ± 266.6	0.045*	806.1 ± 314.0	647.5 ± 265.1	0.022*

Values are mean ± SD or number (%).

*AE*+ patients with dose-related adverse events, *AE− *patients without dose-related adverse events, *WT* body weight, *Dz*+ , patients with only dizziness, *Dz− *patients without only dizziness, *ASMs* antiseizure medications, *Co-ASMs* existence of concomitant ASMs, *Co-EIASMs* existence of concomitant enzyme-inducing ASMs (≤ 6), *OXC* oxcarbazepine, *PK* pharmacokinetics, *MHD* Monohydroxy derivative of oxcarbazepine, *AUC* area under the plasma concentration–time curve.

*P < 0.050.

^a^Values in median [interquartile range].

Statistical analysis: Fisher exact test or Mann–Whitney’s U test.

### Population PK analysis

A one-compartment model with a first-order absorption model and proportional residual error adequately described the MHD concentration–time profiles. We explored several covariates, including age, body weight, sex, and concomitant drugs. The only covariate incorporated for CL/F and V/F was body weight. The use of EIASMs (carbamazepine, phenytoin, phenobarbital, or valproic acid) was tested as a covariate, and CL/F increased 7% in the patients using EIASMs. However, incorporation of the use of EIASMs as a covariate did not improve the model based on OFV and the goodness-of-fit plot; thus, the use of EIASMs was not included as a covariate in the final model. The final parameter estimates for CL/F, V/F, and ka were 1.65 L/h, 59.0 L, and 0.34 h^−1^, respectively (Table 2). The basic goodness-of-fit plots (Supplementary Fig. 1) showed no pronounced bias, which means that the final model adequately described the data.

**Table 2:** Parameter estimates and variability for the population pharmacokinetic model of MHD.

Parameters	Estimates (RSE^a^)	Bootstrap median (95% CI)
**Structural model**		
CL/F = θ1 × (WT/66)^θ2^ ; apparent clearance (L h^−1^)		
θ1; CL/F typical value (L h^−1^)	1.65 (1.8%)	1.65 (1.59–1.70)
θ2; body weight exponent	0.67 (14.2%)	0.66 (0.47–0.85)
Vd/F (L) = θ3 × (WT/66)^θ4^; apparent volume of distribution (L)		
θ3; Vd/F typical value (L h^−1^)	59.0 (4.5%)	59.2 (54.2–64.8)
θ4; body weight exponent	0.96 (18.1%)	0.95 (0.59–1.3)
ka; absorption rate constant (h^−1^)	0.34 (9.7%)	0.34 (0.28–0.41)
**Inter-individual variability (IIV)**		
IIV CL/F (%CV)	29.2 (5.4%)	29.7 (26.2–32.9)
IIV V2/F (%CV)	41.5 (14.3%)	42.5 (29.8–57.3)
**Residual error**		
Proportional error (SD; ng mL^−1^)	0.13 (10.8%)	0.13 (0.10–0.16)

^a^CI: confidence interval, which was estimated by applying the final population pharmacokinetic model to 1000 resampled datasets.

*CV* coefficient of variation, *MHD* monohydroxy derivative of oxcarbazepine, *RSE* relative standard error, *SD* standard deviation, *WT* body weight.

**Figure 1:**
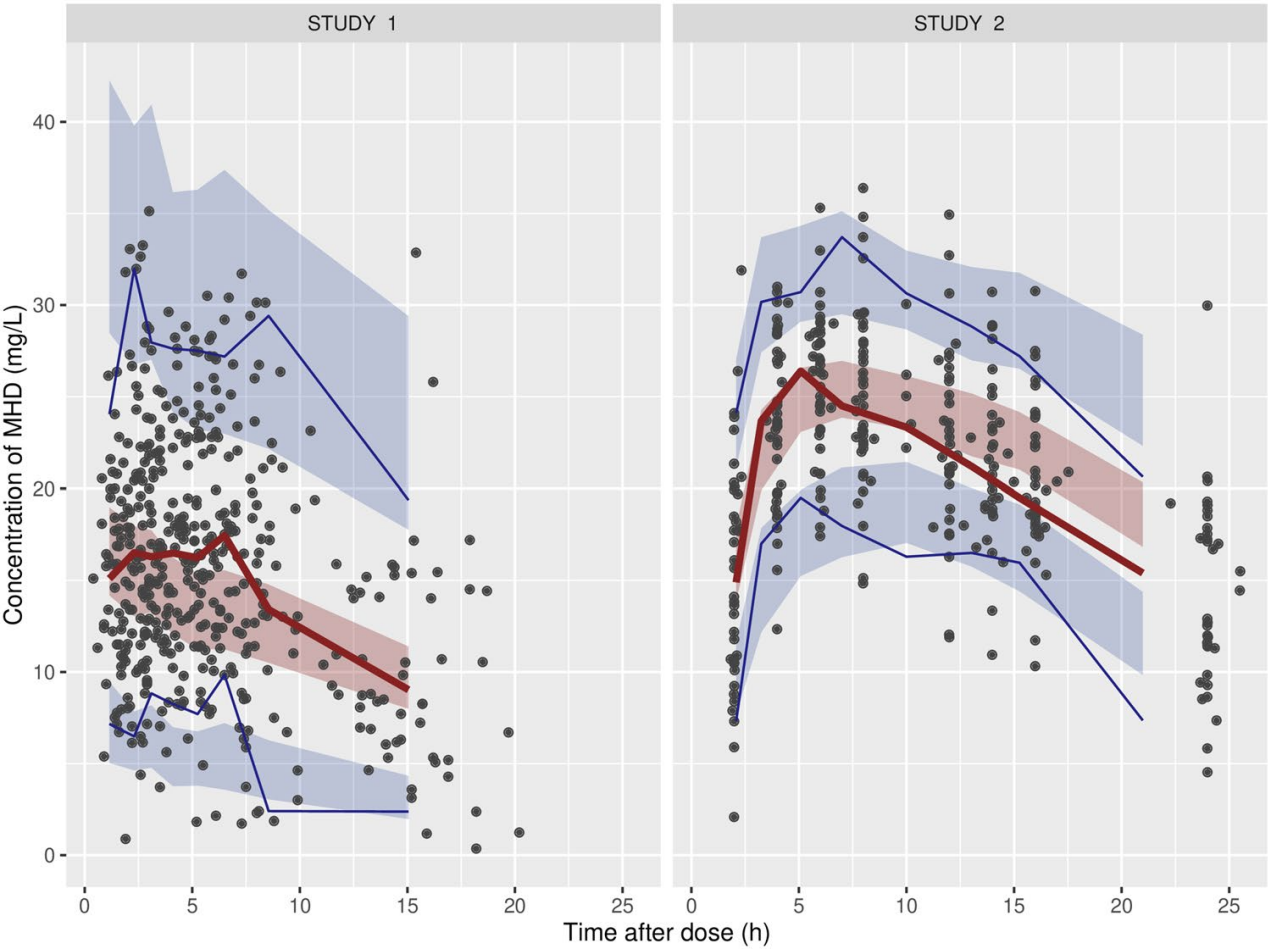
Visual predictive check for MHD. The shaded areas represent the 5th, 50th and 95th percentiles of simulated data (n = 1000). The solid lines are the median along with the 5th and 95th percentiles of the observed data (n = 1000). The left panel shows the results for Study 1, and the right panel shows the results for Study 2. Study 1 – Epilepsy Registry Cohort; Study 2 – Sigle dose study of oxcarbazepine.

The median parameter estimates obtained from the bootstrap analysis were similar to those of the final model, indicating acceptable precision of the final model (Table 2). In visual predictive checks, the median and 90% interpercentile range curves of the simulated concentrations adequately predicted the PK profiles of MHD (Fig. 1).

### Adverse events of oxcarbazepine

A total of 33 patients (7.38%) experienced at least one adverse event during OXC administration. Dizziness (24, 72.7%) was the most common symptom among the adverse events, followed by hyponatremia (4, 12.1%), which showed 129.3 mEq/L on average (range 126 to 131). Three patients (9.09%) suffered from somnolence. Otherwise, skin rash, diplopia, and headache were shown in two patients (6.06%). Tremor, dysarthria, and epistaxis were also reported in one patient (3.03%).

### Association of oxcarbazepine adverse events with MHD level

To elucidate the relationship between OXC and MHD levels and OXC adverse events, we analysed the groups that had adverse events. Adverse events were defined into two groups: the group with DRAEs, including dizziness, somnolence, headache, or diplopia, and the group who reported only dizziness (Table 1). For the patients with DRAEs (n = 28), the OXC dose and MHD trough level showed a tendency to be higher than those of the patients without DRAEs, but the difference was not statistically significant (1130 vs. 990, *p* = 0.181, 15.24 vs. 12.58, *p* = 0.086, respectively). However, the MHD area under the plasma concentration–time curve (AUC) was significantly higher in the DRAE group (753.1 vs. 647.2, *p* = 0.045). MHD trough and MHD AUC showed a significant association with dizziness adverse events (17.48 vs. 12.55, *p* = 0.013, 806.1 vs. 647.5, *p* = 0.022, respectively). The OXC dose was also higher in the patients who showed only dizziness, but it did not reach statistical significance (1216 vs. 989, *p* = 0.053). No significant differences were found when comparing the levels of co-administered ASMs between groups, respectively, depending on the presence of OXC AEs, DRAEs, or dizziness alone (Supplementary Table).

**Figure 2:**
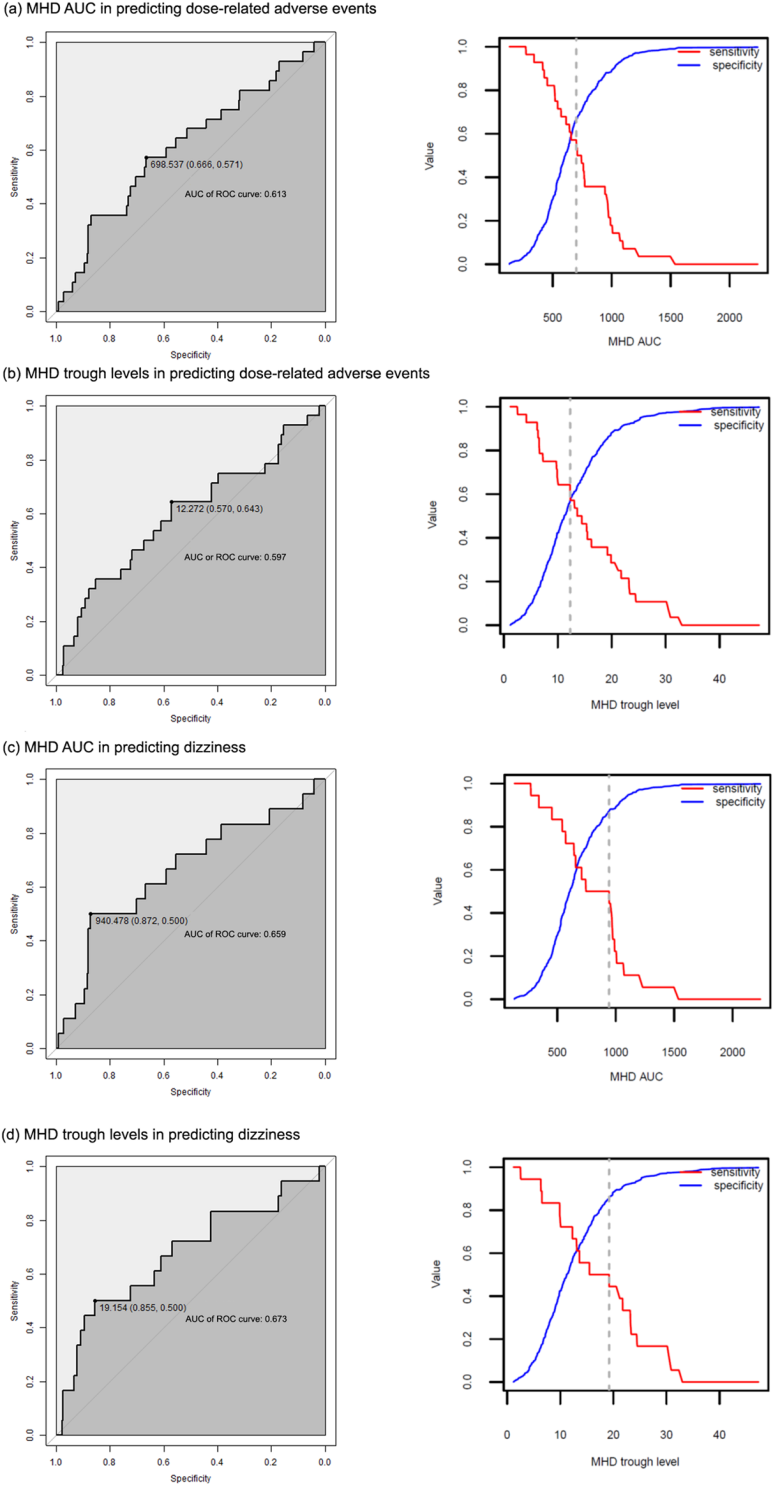
ROC curve for MHD levels in predicting adverse events of oxcarbazepine. (**a**) MHD AUC in predicting dose-related adverse events; (**b**) MHD trough levels in predicting dose-related adverse events; (**c**) MHD AUC in predicting dizziness occurrence; (d) MHD trough levels in predicting dizziness occurrence; ROC, receiver-operator characteristics; AUC, area-under the curve. The data are presented as MHD levels (specificity, sensitivity). Left panels show the ROC curves and right panels show the sensitivity and specificity curve. *AUC represented in each panel is area-under the curve calculated from the ROC curve, not the MHD AUC.

Using ROC curves, we estimated the cut-off value of MHD for the occurrence of adverse events. For DRAEs, the cut-off values of MHD AUC and trough levels were 698.5 mg h/L (specificity, sensitivity 0.571) and 12.27 mg/L (specificity 0.570, sensitivity 0.643), respectively (Fig. 2a,b). For dizziness occurrence, the cut-off values of MHD were higher than those for DRAE; the MHD AUC level was 940.5 mg h/L (specificity 0.872, sensitivity 0.500), and the trough level was 19.15 mg/L (specificity 0.855, sensitivity 0.500) (Fig. 2c,d).

Multivariate analysis was performed to validate the association of the MHD trough and MHD AUC with adverse events (Table 3). Though the MHD trough level was not qualified for the statistical significance in the univariate analysis of the DRAE group (*p* = 0.086), we included it in the multivariate analysis to compare with the result of the sole dizziness group. Since OXC and its derivative are mainly excreted by the kidney, the glomerular filtration rate was also considered in the analysis. In the DRAE group, neither the MHD trough nor the MHD AUC showed a significant association (*p* = 0.056 and *p* = 0.062, respectively). However, the sole dizziness symptom showed a significant association with both MHD trough and MHD AUC (*p* = 0.013, OR 1.079, 95% CI 1.016–1.145, *p* = 0.038, OR 1.002, CI 1.000–1.1003, respectively).

**Table 3:** Multivariable analysis adjusted for age, sex, and GFR.

	Dose-related adverse events (n = 28)				Only dizziness (n = 18)			
	OR	*P*-value	OR	*P*-value	OR	*P*-value	OR	*P*-value
Sex	1.001	0.968	1.001	0.967	0.976	0.268	0.977	0.275
Age	1.202	0.689	1.214	0.672	0.921	0.887	0.984	0.978
GFR	1.002	0.877	1.001	0.929	0.989	0.378	0.988	0.359
MHD trough	1.051	0.056			1.079 (1.016–1.145)^a^	0.013*		
MHD AUC			1.001	0.062			1.002 (1.000–1.003)^a^	0.038*

*GFR* glomerular filtration rate, *MHD* Monohydroxy derivative of oxcarbazepine, *AUC* area under the plasma concentration–time curve.

*P < 0.050.

^a^95% confidence interval.

## Discussion

We developed a population PK model of MHD, the major metabolite of OXC in patients with epilepsy. In addition, the trough level and AUC were estimated from the final PK model to analyse the relationship between PK variables and adverse events of OXC.

As with previous population PK models^10,19–23^, we developed a population PK model using MHD serum concentrations. Some studies included OXC itself as well as MHD in model development^21,22^, but OXC is rapidly absorbed and almost completely converted into MHD, which is the major active metabolite in the human body. There are no data comparing potency in human, as it is difficult to compare the potency of MHD and OXC due to rapid absorption of OXC and its conversion to MHD. However, an in vivo study has shown that the two have similar potency^24^. Moreover, exposure to OXC is negligibly small compared to MHD, so our study did not include OXC in the model development.

Body weight was incorporated as the only covariate for CL/F and V/F in our model. In most studies, the use of EIASMs (carbamazepine, phenytoin, phenobarbital, and/or valproic acid) was a major covariate, showing increased CL/F from 17 to 30% in patients with EIASMs^10,19,22,23^. However, CL/F increased 7% in our study when incorporated as a covariate, and it did not significantly improve the model based on several diagnostic methods, such as OFV change and goodness-of-fit plots. According to in vitro and in vivo studies, OXC and MHD are competitive inhibitors of CYP2C19 and inducers of CYP3A4/5, which can lead to changes in plasma concentrations of other drugs, whereas the plasma concentrations of OXC and MHD can be affected by EIASMs^6,9,10^. However, the use of EIASMs did not show a significant difference in exposure in our study, which means that EIASMs may not actually have a decisive effect on the dose of OXC and MHD in the clinical setting.

While most studies used the population PK model to explore PK characteristics of OXC and MHD and analysed drug interactions with other ASMs, we investigated the relationship of the MHD level and the adverse events of OXC. The DRAEs showed a tendency to have higher MHD trough levels and AUCs but were not statistically significant. On the other hand, the sole dizziness symptom showed a significant correlation with both the MHD trough level and AUC. This result implies that the sole dizziness symptom could be associated with the MHD level in a dose-dependent manner. Moreover, using ROC curves, the cut-off value of MHD was also estimated for the occurrence of adverse events. For example, in Fig. 2d, the MHD trough level would be higher than 19.15 mg/L (cut-off level) for 50.0% of the patients who have sole dizziness and less than 19.15 mg/L for 85.5% of the patients without dizziness. It is meaningful that our study quantitatively evaluated the relationship between drug serum levels and the DRAEs of OXC, which has never been rigorously validated before.

Dizziness is a complex term of various subjective symptoms, including “vertigo”, “light-headedness”, “unsteadiness”, and “wooziness”, reported by patients with drug adverse events. To differentiate the complaint into a homogenous feature, we focused on “wooziness”, excluding vertigo, light-headedness, and unsteadiness, which were accompanied by diplopia, somnolence, and tremor. Patients with OXC-induced hyponatremia also experience dizziness^11^, so patients who suffered from both dizziness and hyponatremia were excluded. In this respect, the sole dizziness reported by the patients in our study might reflect an aspect of the pure symptom “wooziness” induced by OXC overdose.

Our study has several limitations in that the number of patients was too small to evaluate the relationship between symptoms of DRAEs other than dizziness and MHD serum levels. Additionally, since this study was based on a retrospective medical review which did not use a structured questionnaire, adverse events might not be fully reported by the patients despite a routine check at our neurology clinic. Further research applying a structured questionnaire would be valuable for a more accurate assessment. During the prospective blood sampling of the patients, the ASM dosage timing varied from patient to patient, and the interval between ASM dosage time and blood sampling was not controlled. Nevertheless, the population PK model adequately described the PK of OXC in patients with epilepsy using sparse sampling, which is closely related to data from real practice.

In conclusion, we developed a new population PK model using sparse sampling data from patients with epilepsy. Our model better reflects the actual clinical situation than the previous model and is useful for choosing an appropriate dosage regimen for patients with epilepsy. In particular, the cut-off value of the MHD serum concentration by sparse samples would be helpful to assess the DRAEs of OXC in practice. We anticipate that further studies of the PK variable will also help in the DRAE monitoring of new ASMs.

## Supplementary information

Supplementary information 1.

Supplementary information 2.

## Data Availability

The data that support the findings of this study are available from the corresponding author upon reasonable request.
