# Supplementary material for: Population pharmacokinetic model development and its relationship with adverse events of oxcarbazepine in adult patients with epilepsy
Source: Sci Rep. 2021 Mar 18;11:6370. doi: 10.1038/s41598-021-85920-0 (PMC7973549; doi:10.1038/s41598-021-85920-0)
Supplement: Supplementary file 1 — Supplementary information 1. [file 41598_2021_85920_MOESM1_ESM.docx]

**Population pharmacokinetic model development and its relationship with adverse events of oxcarbazepine in adult patients with epilepsy**

**Running head:** Population pharmacokinetic model of oxcarbazepine and its adverse events

Yoonhyuk Jang^1†^, Seonghae Yoon^2,3†^, Tae-Joon Kim^4†^, SeungHwan Lee^3^, Kyung-Sang Yu^3^, In-Jin Jang^3^, Kon Chu^1*^, Sang Kun Lee^1*^

*^1^Department of Neurology, Laboratory for Neurotherapeutics, Comprehensive Epilepsy Center, Biomedical Research Institute, Seoul National University Hospital, Seoul, South Korea*

*^2^Clinical Trials Center, Seoul National University Bundang Hospital, Seoul, Korea*

*^3^Department of Clinical Pharmacology and Therapeutics, Seoul National University College of Medicine and Hospital, Seoul, Korea*

*^4^Department of Neurology, Ajou University School of Medicine, Suwon, South Korea*

^†^ These authors contributed equally to this study as co-first authors.

^*^ These authors contributed equally to this study as co-corresponding authors.

*Character count for the title: 134*

*Character count for the running head: 72*

*Total word count for the abstract: 196*

*Total word count for the manuscript: 2573*

*Number of references: 21*

*Number of tables: 3*

*Number of figures: 2*

*Number of supplementary figures: 1*

**Correspondence:**

Kon Chu, MD, PhD

Department of Neurology, Seoul National University Hospital,

101 Daehak-ro, Jongno-gu, Seoul 110-744, South Korea

Tel.: +82-2-2072-1878/Fax: + 82-2-3672-7553

Email: [stemcell.snu@gmail.com](mailto:stemcell.snu@gmail.com)

and

Sang Kun Lee, MD, PhD

Department of Neurology, Seoul National University Hospital,

101 Daehak-ro, Jongno-gu, Seoul 110-744, Korea

Tel: +82-2-2072-2923/Fax: +82-2-3672-7553

E-mail: [sangkun2923@gmail.com](mailto:sangkun2923@gmail.com)

**Supplementary figure 1. Basic goodness-of-fit plots of final model.** (a) Observed values versus individual predicted values; (b) observed values versus population predicted values; (c) conditional weighted residuals (CWRES) versus population predicted values; and (d) CWRES versus time. Black and grey solid lines indicate the line of identity and Loess (locally weighted smoothing), respectively.

| **Supplementary Table. The dose and level of co-administered ASMs according to the adverse events of OXC** | | | | | | | | | | |
| --- | --- | --- | --- | --- | --- | --- | --- | --- | --- | --- |
| Anti-seizure medications | Item | All adverse events | | | Dose-related adverse events | | | Only dizziness | | |
|  |  | Present (n=31) | Absent (n=416) | P-value | Present (n=28) | Absent (n=419) | P-value | Present (n=18) | Absent (n=429) | P-value |
| Valproate | n | 6 (19%) | 56 (13%) |  | 5 (18%) | 57 (14%) |  | 3 (17%) | 59 (14%) |  |
|  | dose | 1250±612 | 1065±415 | 0.609 | 1300±671 | 1064±413 | 0.560 | 1333±577 | 1069±426 | 0.480 |
|  | level^*^ | 59.9±29.8 | 59.5±26.3 | 0.880 | 60.5±33.2 | 59.4±26.0 | 0.822 | 59.8±39.2 | 59.5±26.0 | 0.853 |
|  | toxic^†^ | 0 | 2/56 (4%) | - | 0 | 2/57 (4%) | - | 0 | 2/59 (3%) | - |
| Phenobarbital | n | 3 (10%) | 7 (2%) |  | 2 (7%) | 8 (2%) |  | 0 | 10 (2%) |  |
|  | dose | 80±35 | 73±33 | 0.859 | 60 | 76±34 | 0.434 |  |  | - |
|  | level | 12.5±2.7 | 14.4±8.1 | 0.833 | 13.7±2.4 | 13.9±7.7 | >0.999 |  |  | - |
|  | toxic | 0 | 0 | - | 0 | 0 | - | 0 | 0 | - |
| Levetiracetam | N | 11 (35%) | 150 (36%) |  | 10 (36%) | 151 (36%) |  | 5 (28%) | 156 (36%) |  |
|  | dose | 1409±625 | 1394±736 | 0.673 | 1450±643 | 1391±734 | 0.549 | 1300±447 | 1398±735 | 0.951 |
|  | level | 15.7±9.1 | 15.6±11.7 | 0.574 | 15.9±9.5 | 15.6±11.7 | 0.566 | 13.9±11.4 | 15.7±11.6 | 0.830 |
|  | toxic | 0 | 3/150 (2%) | - | 0 | 3/151 (2%) | - | 0 | 3/156 (2%) | - |
| Lamotrigine | n | 4 (13%) | 37 (9%) |  | Same result as left box | | | 3 (17%) | 38 (9%) |  |
|  | dose | 213±85 | 249±102 | 0.596 |  |  |  | 183±76 | 250±101 | 0.320 |
|  | level | 4.85±2.13 | 5.27±3.11 | 0.916 |  |  |  | 3.97±1.45 | 5.33±3.09 | 0.457 |
|  | toxic | 0 | 1/37 (3%) | - |  |  |  | 0 | 1/37 (3%) | - |
| Pregabalin | n | 4 (13%) | 50 (13%) |  | Same result as left box | | | 1 (6%) | 56 (13%) |  |
|  | dose | 413±130 | 275±140 | 0.019* |  |  |  | 300 | 284±144 | 0.807 |
|  | level | 3.23±2.00 | 2.75±1.77 | 0.599 |  |  |  | 0.742 | 2.82±1.77 | 0.222 |
|  | toxic | 0 | 0 | - |  |  |  | 0 | 0 | - |

^*^Levels of antiseizure medications are expressed in μg/mL.

^†^Toxic levels of antiseizure medications: valproate >100, Phenobarbital >40, Levetiracetam > 46, Lamotrigine >15, and Pregabalin >10

^‡^Levels of the other ASMs not shown in the Supplementary Table were not measured.
