# Supplementary material for: Population pharmacokinetic model development and its relationship with adverse events of oxcarbazepine in adult patients with epilepsy
Source: Sci Rep. 2021 Mar 18;11:6370. doi: 10.1038/s41598-021-85920-0 (PMC7973549; doi:10.1038/s41598-021-85920-0)
Supplement: Supplementary file 2 — Supplementary information 2. [file 41598_2021_85920_MOESM2_ESM.docx]

**Population pharmacokinetic model development and its relationship with adverse events of oxcarbazepine in adult patients with epilepsy**

**Running head:** Population pharmacokinetic model of oxcarbazepine and its adverse events

Yoonhyuk Jang^1†^, Seonghae Yoon^2,3†^, Tae-Joon Kim^4†^, SeungHwan Lee^3^, Kyung-Sang Yu^3^, In-Jin Jang^3^, Kon Chu^1*^, Sang Kun Lee^1*^

*^1^Department of Neurology, Laboratory for Neurotherapeutics, Comprehensive Epilepsy Center, Biomedical Research Institute, Seoul National University Hospital, Seoul, South Korea*

*^2^Clinical Trials Center, Seoul National University Bundang Hospital, Seoul, Korea*

*^3^Department of Clinical Pharmacology and Therapeutics, Seoul National University College of Medicine and Hospital, Seoul, Korea*

*^4^Department of Neurology, Ajou University School of Medicine, Suwon, South Korea*

^†^ These authors contributed equally to this study as co-first authors.

^*^ These authors contributed equally to this study as co-corresponding authors.

*Character count for the title: 134*

*Character count for the running head: 72*

*Total word count for the abstract: 196*

*Total word count for the manuscript: 2992*

*Number of references: 23*

*Number of tables: 3*

*Number of figures: 2*

*Number of supplementary figures: 1*

*Number of supplementary tables: 1*

**Correspondence:**

Kon Chu, MD, PhD

Department of Neurology, Seoul National University Hospital,

101 Daehak-ro, Jongno-gu, Seoul 110-744, South Korea

Tel.: +82-2-2072-1878/Fax: + 82-2-3672-7553

Email: [stemcell.snu@gmail.com](mailto:stemcell.snu@gmail.com)

and

Sang Kun Lee, MD, PhD

Department of Neurology, Seoul National University Hospital,

101 Daehak-ro, Jongno-gu, Seoul 110-744, Korea

Tel: +82-2-2072-2923/Fax: +82-2-3672-7553

E-mail: [sangkun2923@gmail.com](mailto:sangkun2923@gmail.com)

**Supplementary Material. NONMEM code for the final model**

$SUBROUTINE ADVAN2 TRANS2

$PK

TVCL = THETA(1)

TVV = THETA(2)

TVKA = THETA(3)

CL = TVCL * EXP(ETA(1))*(BW/66)**THETA(4)

V = TVV * EXP(ETA(2))*(BW/66)**THETA(5)

KA = TVKA * EXP(ETA(3))

K=CL/V

SC = V/1000

$ERROR

IPRED = F

W = SQRT(THETA(6)**2 + THETA(7)**2 * IPRED**2)

IRES = DV - IPRED

IWRES = IRES/W

Y = IPRED + W * EPS(1)

$THETA

(0, 1.67) ; CL

(0, 64.6) ; V

(0, 0.447) ;KA

(0, 0.696) ;CL-WT

(0, 1.12) ;V-WT

(0.0001) FIX ; ADD

(0, 0.179) ; PROP

$OMEGA

0.0801 ; CL

0 FIX ; V

0.1 ; KA

$SIGMA

1 FIX

$ESTIMATION MAX=9999 SIG=3 METHOD=1 INTER PRINT=10 NOABORT

$COV
